# The Management of Intussusception: A Systematic Review

**DOI:** 10.7759/cureus.49481

**Published:** 2023-11-27

**Authors:** Majed Ali Attoun, Shuruq Mousa D Albalawi, Afnan Ayoub, Ali K Alnasser, Esraa H Alkaram, Fouz A Khubrani, Khalid J Alzahrani, Kholoud A Alatawi, Nura Almutairi, Almuhannad G Alnami

**Affiliations:** 1 Department of Surgery, King Salman Armed Forces Hospital, Tabuk, SAU; 2 Faculty of Medicine, University of Tabuk, Tabuk, SAU; 3 Faculty of Medicine, Batterjee Medical College, Jeddah, SAU; 4 Faculty of Medicine, Medical University of Lodz, Lodz, POL; 5 Department of Emergency Medicine, Almana Group of Hospitals (AGH), Dammam, SAU; 6 Faculty of Medicine, King Abdulaziz University, Jeddah, SAU; 7 Department of Radiology, Imam Abdulrahman Bin Faisal University, Dammam, SAU; 8 Department of General Surgery, King Khalid Hospital, Tabuk, SAU; 9 Faculty of Medicine, Jazan University, Jazan, SAU

**Keywords:** covid-19, ultrasound, intussusception, fluoroscopy, enema, child, adult

## Abstract

Intussusception (ISN) is a dangerous condition where a portion of the intestine slides into an adjacent area of the intestine. This telescoping motion frequently prevents liquids or food from flowing through. Developing management guidelines for ileocolic (IC) intussusception was the aim of this systematic study. Data sources were PubMed/Medical Literature Analysis and Retrieval System Online (MEDLINE), Scopus, and Embase databases. Our review investigated English-language articles (from 2010 to 2023) according to the Preferred Reporting Items for Systematic Reviews and Meta-Analyses (PRISMA) guidelines. Overall, there were 15 articles. Surveys and analyses of national databases were the most widely used methods (n=15). The search identified 561 studies; 15 were eligible for inclusion in the analysis. Further understanding of the management of intussusception may help improve evaluation and management in the future. The use of preventive antibiotics does not reduce problems following radiologic reduction. When clinically appropriate, repeated attempts at enema reduction may be made. After the enema reduction of ileocolic intussusception, patients can be safely watched in the emergency room (ER), thereby avoiding hospitalization. Success rates for laparoscopic reduction are high. When it comes to intussusception in children who are hemodynamically stable and do not have a serious illness, there is no need for pre-reduction antibiotics. Prioritizing nonoperative outpatient (OP) therapy is recommended as the primary approach, with the utilization of minimally invasive procedures to avoid the necessity for laparotomy. The management of colonic intussusception involves complete removal in one piece, while enteric intussusception can be addressed through reduction followed by resection. A targeted approach is recommended, recognizing the intermediate forms of intussusception that may exist between the colonic and enteric types. It is essential to note that the prevailing treatment for adult intussusception remains to be surgical intervention.

## Introduction and background

Ileocolic (IC) intussusception (ISN) presents as a frequent abdominal emergency in children under three years old, with an incidence ranging from 0.33 to 0.71 per 1,000 person-years. Surgical resection is necessary in only a small percentage of cases, as the majority originates benignly without a pathological lead point. Practices regarding preventive antibiotics, radiologic reduction procedures, postreduction care, and surgical techniques vary across institutions [1,2].

Intussusception is a disorder characterized by the folding of a portion of the intestine into the adjacent tract. While large bowel involvement is uncommon, it predominantly affects the small bowel. Symptoms include abdominal pain with fluctuations, vomiting, bloating, and the presence of bloody feces. Small bowel blockage is a potential outcome, with risks of bowel perforation or peritonitis [3].

The cause of intussusception remains unknown, with around 90% of pediatric cases having an enigmatic origin. Possible contributors include abnormal motility, anatomical factors, and infections. Known causes encompass infections, anatomical factors, altered motility, Meckel's diverticulum, duplication, polyps, appendicitis, hyperplasia of Peyer patches, and idiopathic causes [4-6].

While there is no firm connection between current rotavirus vaccines and intussusception, a previous version, no longer in use, was theorized to be associated with the condition. Intussusception is typically diagnosed in early childhood, with approximately 2,000 infants in the United States experiencing it within their first year of life. Onset usually occurs at five months of age, peaking between four and nine months and gradually declining by 18 months. Males are more susceptible, with a ratio of approximately 3:1. In adults, intussusception is linked to neoplasia and accounts for about 1% of intestinal blockages [7,8].

Under normal circumstances, the ileum enters the cecum, and instances of the ileum or jejunum prolapsing into itself are exceedingly rare. During an intussusception, the intussusceptum, or the part that prolapses into the other, is typically located near the intussuscipiens. This occurs due to the peristaltic motion of the intestine, drawing the proximal segment into the distal section. About 10% of intussusceptions involve an anatomical lead point [9].

Ischemia occurs when the blood supply to the trapped portion of the colon is cut off. The mucosa, being susceptible to ischemia, reacts by sloughing off into the stomach. This process leads to the sloughing of mucosa, blood, and mucus, resulting in feces with a characteristic appearance similar to "red currant jelly." The presence of "red currant jelly"-like feces is a rare symptom of intussusception and should be considered during the diagnosis of children who pass any kind of bloody stool [10].

Usually, intussusception does not pose an immediate risk to life. Barium, water-soluble, and air-contrast enemas are typically effective treatments that not only confirm the diagnosis but also successfully alleviate the condition. The success rate exceeds 80%. However, recurrence may occur in up to 10% of cases within a day [11,12].

Surgery is necessary for cases that cannot be reduced nonsurgically. During surgical reduction, the telescoped component is physically manipulated by the surgeon. If the surgeon cannot successfully reduce it, the affected area is surgically removed. A laparoscopic procedure, using forceps to separate the intestinal segments, can also be employed to reduce an intussusception. The differential diagnosis includes gastric volvulus, internal hernias, testicular torsion, volvulus, colonic issues, cyclic vomiting syndrome, emergency treatment of gastroenteritis, intestinal hernias, appendicitis, and obvious abdominal trauma in emergency medicine [13,14].

Various medications, including dexamethasone and glucagon, have been studied to improve the success rate of enema reduction. The addition of dexamethasone has shown promising results. However, there are insufficient data to support the idea that the addition of glucagon significantly increases the success rate of enema reduction [15].

The consequences of intussusception, such as perforation, intestinal necrosis, and, infrequently, short bowel syndrome, require prompt management. On the other hand, postoperative intussusception (POI) may develop as an uncommon side effect after various surgical procedures, including pancreatectomy, Ladd operation, diaphragmatic surgery, and retroperitoneal tumor resection. There is a 0.25% chance of postoperative infection in children after a laparotomy [16].

This study thoroughly evaluated the latest research on the treatment of adult and pediatric intussusception. The predetermined areas of interest included the use of minimally invasive procedures, radiologic management, antibiotic stewardship, and emergency department (ED) discharge. The results of this systematic review were compiled to develop an evidence-based management algorithm suitable for routine use across various hospital settings.

## Review

Methods

Following the guidelines set by the Preferred Reporting Items for Systematic Reviews and Meta-Analyses (PRISMA), we developed the procedures for the current systematic review.

Search

**Figure 1:**
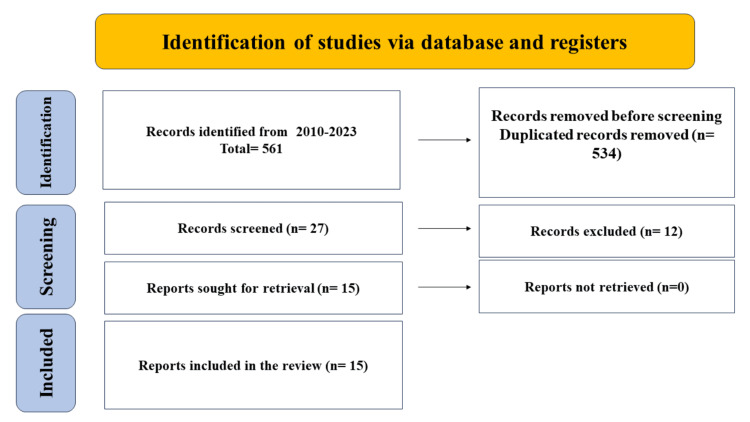
Study flow diagram (Preferred Reporting Items for Systematic Reviews and Meta-Analyses {PRISMA})

For this review, keywords and Medical Subject Heading phrases were used to search PubMed/Medical Literature Analysis and Retrieval System Online (MEDLINE) (2010-August 2023) and Embase (2010-August 2023) for five important concepts: intussusception, causes, radiologic management, antibiotic stewardship, and surgical management. Limits were applied so that only English-language items were included. Examining the references found in the identified papers led to the creation of additional studies. After retrieving all full texts, 15 articles were discovered to be part of this review. The Preferred Reporting Items for Systematic Reviews and Meta-Analyses (PRISMA) technique was used for search screening and article shortlisting (Figure 1).

The inclusion criteria include English articles and articles and reviews demonstrating intussusception and possible management options.

The exclusion criteria encompassed incomplete data presentation, with research data before 2010 being excluded. The data were excluded if they did not discuss intussusception.

Data collection

Twenty-seven papers were included after titles and abstracts were checked out. Of the 561 studies that were found through the search, 15 papers were qualified for analysis after full-text reading (Figure 1).

Data extraction

Table 1 provides a brief description of the data, which include the author(s) name and year, study aim, and finally conclusion of the study.

**Table 1:** Brief description of the data, which includes the author(s) name and year, the study aim, and finally the conclusion of the study IC, ileocolic; B-USGHE, B-ultrasound-guided hydrostatic enema

Serial number	Author and year	Aim of the study	Conclusion
1	Gluckman et al., 2017 [13]	We found six randomized trials with 822 participants in total that examined the treatment of intussusception in children and evaluated various therapeutic modalities. Three active trials were also recognized.	The primary outcome was that a number of children had successfully decreased intussusception. In addition, a number of kids returned with recurrent intussusceptions and an assessment of the harms (adverse events) brought on by the therapies. Data from two trials indicate that utilizing air instead of liquid during an enema reduces the risk of intussusception. Additionally, data from two studies indicate that, regardless of whether liquid or air is used for the enema, giving the kid with intussusception a steroid medication, such as dexamethasone, may lessen the likelihood that the condition would return. On intraoperative and postoperative complications, as well as other adverse events, we could only find scant information.
2	Ito et al., 2012 [17]	The literature was meticulously gathered over the Internet using the keywords "children" and "intussusception." The Oxford Centre for Evidence-Based Medicine's levels of evidence were used to grade the quality of evidence in each study. Fifty clinical questions and their corresponding answers made up the guidelines. The protocols that were advised based on the degrees of evidence strength were given recommendation grades. To promote early diagnosis, proper treatment selection, and patient transfer for referral to a tertiary hospital in cases of severe illness, three criteria were proposed: "diagnostic criteria," "severity assessment criteria," and "criteria for patient transfer."	Recommendation regarding barium is no longer advised for enema reduction since the patient becomes critically unwell as soon as a perforation occurs. Under either fluoroscopic or sonographic direction, the use of other contrast media, such as water-soluble iodinated contrast, normal saline, or air, is advised. If the patient's status is stable and the initial enema only partially decreased the intussusception, a delayed repeat enema is advised since it improves the reduction success rate.
3	Tarchouli and Ait Ali, 2021 [18]	This is a retrospective analysis of adult patients who were surgically treated at our facility between January 2009 and December 2018 and who had an intestinal intussusception diagnosis. Data on histology, surgery, and clinical conditions were gathered and examined.	Any patient presenting with subacute abdominal pain should be evaluated for adult intussusception (AI). For colonic intussusceptions, intestinal resection without attempting reduction is strongly recommended due to the high probability of cancer. However, for intestinal intussusceptions, a more targeted strategy may be used.
4	Mittal et al., 2023 [19]	Records pertaining to intussusceptions diagnosed between July 2002 and September 2014 were assessed with respect to patient age, sex, clinical observations, time of admission, results of ultrasonography, treatment strategies, and outcomes.	Surgery is required for intussusception patients if there is peritoneal irritation. Otherwise, hydrostatic or pneumatic reduction is recommended in individuals with IC intussusception who do not exhibit any signs of peritoneal irritation. The next course of action is surgery if this fails. Shorter than 2.3 cm and free of peritoneal irritation, small bowel intussusceptions (SBIs) typically disappear on their own. Spontaneous reduction of longer than 4 cm is uncommon for individuals, especially in patients who have had previous abdominal surgery.
5	Xia et al., 2021 [20]	To help surgeons better manage patients who encounter difficulties from postoperative intussusception, this study aims to provide an overview of the practical experience with this condition. Furthermore, we anticipate that our results will direct further studies on this subject.	Intussusception after gastrectomy is a rare but challenging condition, often subacute and unaffected in children. Diagnosis is often delayed or missed. Abdominal computed tomography (CT) is the most sensitive examination for intussusception diagnosis, determining its location and presence. Patients with persistent abdominal pain after gastrectomy should consider small bowel intussusception. Early identification through abdominal CT can help achieve timely treatment and avoid missed diagnoses. Surgical intervention is mandatory due to increased mortality after over 48 hours of intussusception. Treatment typically involves manual reduction, and if necrosis is found, the necrotic bowel segment can be removed locally.
6	Ye et al., 2019 [21]	Up to August 2018, the PubMed, Embase, and Cochrane databases were searched in our systematic review. The main result was the chance ratio for each of the following possible risk factors: pathological lead point (PLP), vomiting, fever, sex, blood in the stool, abdominal pain, and right abdominal mass.	This meta-analysis's primary finding was that children who had both fever and PLP would be more likely to experience a recurrence of intussusception after enema reduction. Recurrent intussusception (RI) patients were shown to have a decreased frequency of vomiting than non-RI patients (control group).
7	Clark et al., 2019 [22]	Before rotavirus vaccinations were developed, we calculated incidence rates, age distributions, and case-fatality ratios (CFRs) for hospital admissions related to intussusception in children under the age of five. All the studies found in a systematic review published between January 2002 and January 2018 were included, and the authors were contacted to obtain more detailed unpublished data on age distributions.	The epidemiology of intussusception differs by nation and location. It will be crucial to comprehend and identify these variations when estimating the possible number of intussusception cases connected to rotavirus vaccinations.
8	Plut et al., 2020 [23]	The most frequent reason for intestinal blockage in young children is intussusception. A crucial part of its diagnosis and care is played by radiology. The evidence that is currently available for optimal practices in radiologic management of pediatric intussusception is compiled in this systematic review.	The preferred imaging modality for diagnosing intussusception is ultrasound (US), due to its high diagnostic accuracy and non-ionizing radiation nature. Both US-guided hydrostatic enema and fluoroscopy-guided pneumatic enema are equally safe and effective methods for reducing intussusception. The effectiveness and safety of the US-guided pneumatic enema, the possible advantages of general anesthesia and sedation for the reduction procedure, and the best way to manage intussusceptions that may involve pathological lead points are among the topics that need more investigation in this area.
9	Sun et al., 2022 [24]	This study examined the epidemiological characteristics of pediatric intussusception, the outcomes of various management approaches, and the variables influencing effective reduction in retrospect.	There was a discrepancy in the age at onset and sex ratio for pediatric intussusception. Some children may be spared radiation exposure due to the successful reduction rate of cleansing enemas for both single and multiple intussusceptions. A successful reduction in cleansing enema was influenced by related factors such as the diameter and length of the intussusception. When it came to successful reduction, there were no appreciable variations between B-USGHE and air enemas. By reducing most recurrent intussusceptions, surgery is still not necessary.
10	Fahiem-Ul-Hassan et al., 2020 [25]	The first management tool for intussusception is currently ultrasound-guided hydrostatic reduction, or HSR. However, HSR faces challenges in addition to the fact that many patients still require surgery as their primary course of treatment for intussusception. This study was conducted to evaluate the effectiveness of HSR and to identify the factors that require surgical exploration in intussusception patients.	HSR is a secure and efficient method of treating intussusception. Patients with risk factors, such as delayed presentation, crescent appearance on ultrasonography (USG), and length greater than 10 cm, experience higher failure rates. In addition, the efficacy of HSR in preventing future recurrences and in cases of multiple intussusceptions, small bowel intussusception, and neonatal intussusception is questionable. When possible, laparoscopy should be used in conjunction with laparotomy to treat these patients. Additionally, bowel ischemia and peritonitis should be ruled out clinically and radiologically prior to starting HSR. In cases of suspected bowel ischemia, USG Doppler could be useful.
11	Li et al., 2021 [26]	A distinct benefit in the clinical diagnosis of intussusception is provided by ultrasonography. Our goal was to assess how well ultrasonography could diagnose pediatric intussusception. We looked for studies about the ultrasonographic diagnosis of intussusception in children in the databases of PubMed, Embase, Web of Science, Cochrane Library, and African Journals Online. Ultimately, it was determined that 14 studies (totaling n=2,367) qualified for inclusion.	There were 0.94 (95% confidence interval: 0.91-0.96) and 0.96 (95% confidence interval: 0.93-0.98) for the pooled sensitivity and specificity, respectively. To sum up, ultrasound is a highly sensitive and specific method for diagnosing intussusception.
12	Leiva et al., 2022 [27]	The first documented instance of a lead point with tissue polymerase chain reaction (PCR) confirming COVID-19 positivity was one of two cases of intussusception in COVID-19-positive patients that were examined. These results were compared to a review of the most recent literature. In patients who test positive for COVID-19, intussusception is becoming more common and frequently necessitates surgical intervention.	When COVID-19-related respiratory symptoms are not present, intussusception may be the presenting symptom. When compared to COVID-19-negative intussusception, there also appears to be a trend toward the need for surgical intervention. PCR can be used to confirm that SARS-CoV-2 is present in particular lead points (lymph nodes), which is what causes the intussusception. Given that surgical intervention is more frequently employed for pediatric COVID-19-positive patients exhibiting gastrointestinal symptoms, healthcare providers ought to have a low threshold for suspecting and diagnosing intussusception.
13	Valentini et al., 2016 [28]	Intestinal intussusception in adults is uncommon and frequently a result of a pathological condition, in contrast to pediatric intussusception. Even in cases that are brief and nonobstructive, the number of radiologic diagnoses of intussusception has increased due to the expanding use of multi-detector computed tomography (MDCT) in abdominal imaging. Several features, including the location of intussusception, the intestinal segments affected, and the extent of the intussuscepted bowel, can be usefully determined by MDCT, which is well suited to defining the disease's presence.	Additionally, bowel wall ischemia and perforation, complications of intussusceptions that require immediate surgical referral, can be seen on MDCT. But not every intussusception requires surgical intervention. This paper reviews the current role of magnetic resonance imaging (MRI) in the diagnosis and treatment of adult intussusception. Specifically, we focus on features such as the presence of a leading point that may help in the accurate patient selection process for surgery.
14	Blaker and Anandam, 2017 [29]	The invagination of the rectal wall into the rectum's lumen is known as rectoanal intussusception. Individuals may show up asymptomatic, with incomplete evacuation, incontinence, or constipation. The most reliable method of detection has always been defecography. Defecography by magnetic resonance imaging and dynamic anal endosonography are substitutes for traditional defecography. But neither of these techniques is as sensitive as traditional defecography.	Treatment options include surgical procedures such as rectopexy, stapled transanal rectal resection, and Delorme, as well as conservative/medical treatments such as biofeedback. If other causes of constipation are absent, recent studies following a trial of unsuccessful nonoperative management demonstrate adequate results with operations performed for rectal intussusception with or without rectocele.
15	Wassmer et al., 2020 [30]	We report a rare instance of a patient with multiple intussusceptions who was immunocompromised. A 59-year-old patient with end-stage renal failure from type 1 diabetes who underwent a kidney-pancreas transplant was admitted to our intensive care unit due to septic shock that appeared to have pulmonary origins. She had surgery to treat several abdominal intussusceptions and bilateral pneumonia that were discovered on a thoracoabdominal CT scan. They discovered four intestinal intussusceptions.	Bowel resection was avoided in favor of manual desinvagination. Following surgery, the patient developed a new intestinal blockage that necessitated a second procedure and revealed a recurrence of one intussusception. Despite the need for high-dose noradrenalin due to septic shock, segmental resection was not carried out. A few days later, the patient passed away due to multi-organ failure that she was experiencing. The intussusceptions were caused by multiple adenomas, as an autopsy revealed. This case demonstrates that AI is rarely a spontaneous illness and that treatment plans should take this into consideration. Guidelines are required to improve the management of AI, as there is currently no systematic approach to the field.

Discussion

This review highlights finding the more appropriate management options for intussusception. This systematic review identified 15 studies presenting intussusception. The invasion of one bowel segment into another is known as intussusception. Children with idiopathic ileocolic intussusception frequently receive a nonoperative reduction as treatment. It is more common in the small intestine and rare in adulthood. Bowel obstruction is typically linked to lead point pathology. Lead point pathology is often benign in adult small bowel intussusception (SBI), although malignant cases are more common because of diffuse metastatic disease. Lead point pathology in adult ileocolic and colonic intussusception is primarily primary adenocarcinoma. Usually, intraoperative or cross-sectional imaging is used to make the diagnosis. A rising number of adult abdominal computed tomography (CT)/magnetic resonance imaging (MRI) scans reveal transitory, asymptomatic intussusceptions, which can be seen without medical intervention [31].

A total of 81 patients participated in the study, comprising 52 males and 29 females, with a mean age of 10.6 months. Ileocolic (IC) in 52 cases, ileoileal (IL) in 26 cases, and jejunojejunal (JJ) in three cases were the types of intussusceptions. Of the patients, 19 (23.5%) had surgery. In 45 (55.5%) of the IC instances, hydrostatic reduction was carried out. Small bowel intussusceptions (SBIs), which range in length from 1.8 to 2.3 cm, spontaneously decreased in 17 (21%) of the patients. Every patient who had surgery had an intussusceptum of at least 4 cm. There was a history of abdominal surgery in three of the four cases of intestinal resection [32].

In the study by Clark et al. (2019), 128 articles with 227 national datasets (61 age distributions, 71 incidence rates, and 95 case-fatality ratios {CFRs}) were found. Twenty-nine weeks was the median age of intussusception in Africa (83% of cases in the first year of life), whereas 35 weeks was the median age in the Western Pacific Region (35% of cases in the first year of life). The annual incidence of hospital admissions due to intussusception per 100,000 children under one year of age varied from 34 (13-56) in Africa to 90 (9-380) in the Western Pacific Region. The CFRs in Africa (one death per 10 hospital admissions) and the rest of the globe (less than one fatality per 100-2,000 hospital admissions) were found to differ significantly [22].

The literature review on managing intussusception in children highlights the need to refrain from administering antibiotics prior to reduction, utilize minimally invasive surgical procedures as the primary operative approach, and repeat radiologic reduction attempts to minimize the necessity for surgery. Furthermore, it highlights that while recurrence is uncommon following surgical or radiologic reduction methods, children older than two years old might need more careful monitoring. A management algorithm developed during the evaluation should only be used on children who are healthy and do not have a life-threatening illness. The assessment highlights the need for improved outcomes and the utilization of healthcare [33].

Pediatric intussusceptions are primarily attributed to brief viral infections, causing transient lymphatic engorgement that results in a lead point and subsequent intussusception. Poor outcomes, such as perforation, sepsis, or hospital readmission, are rare, as most children tend to recover well after undergoing enema reduction or surgery. The resolution of intussusception, coupled with recovery from the viral infection, likely eliminates the chance of future recurrence. Nevertheless, the current body of evidence for guiding clinical management lacks sufficient level 1 and level 2 support. Most studies are limited to single-center, small-scale reporting or retrospective investigations. Although the study incorporated larger cohort studies employing administrative claim data, it was constrained by retrospective and nonclinical information [34,35].

Future research can improve the accuracy of assessing healthcare usage by tracking individuals over time through hospitalizations and emergency room (ER) visits using huge datasets. Enrollment in randomized control trials could be difficult because doctors and surgeons are worried about equipoise. On the other hand, since inpatient (IP) surveillance and release from the ER have a big impact on healthcare utilization, the outpatient (OP) management of pediatric intussusception would be a great place to start a trial. Further research on safety, effectiveness, the timing of delayed repeated enemas for radiographic reduction, innovative reduction procedures, and the long-term hazards and benefits of concurrent appendectomy during the operative reduction of intussusception are all areas that warrant attention [33-35].

Ten studies examining outpatient therapy outcomes in individuals aged 0-18 years with intussusception, who underwent effective enema reduction, were included in the analysis. The recurrence rates for inpatient (IP) and outpatient (OP) settings were 6% and 8%, respectively (p=0.20). Similar recurrence rates were observed within 24 hours (IP 1% versus OP 0%, p=0.90) and 48 hours (IP 1% versus OP 2%, p=0.11). The rates of return to the emergency room showed no significant difference (IP 6% versus OP 14%, p=0.11). Additionally, the need for an operation was comparable between the two groups (IP 2% versus OP 1%, p=0.84). Following enema reduction, outpatient care for intussusception resulted in a shorter hospital stay without impacting mortality, recurrence, or the rate of readmission to the emergency room. The meta-analysis's conclusions suggest that outpatient management may be a low-risk alternative, potentially reducing the demand for hospital resources [36].

A total of 83 articles were systematically examined and included in the evaluation. The findings suggest that the use of preventive antibiotics does not alleviate issues following radiologic reduction. In clinically appropriate situations, it is advisable to consider repeated attempts at enema reductions. Subsequent to the enema reduction of ileocolic intussusception, patients can be safely monitored in the emergency room, thus averting the need for hospitalization. Laparoscopic reduction demonstrates consistently high success rates. In the case of intussusception in pediatric patients who are hemodynamically stable and lack serious illness, pre-reduction antibiotics may not be necessary. The optimal course of action is to emphasize nonoperative outpatient therapy. Additionally, minimally invasive procedures prove effective in preventing the need for laparotomy [33].

A total of 40 retrospective case series yielded the identification of 1,219 patients. Colonic intussusception should be removed in one piece, whereas enteric intussusception can be treated via reduction and then resection. A targeted approach is advised due to the intermediate forms of intussusception that exist between colonic and enteric intussusceptions. The standard treatment for adult intussusception is still surgery [37].

In addition to having a low chance of developing ISN, children infected with SARS-CoV-2 may also require mechanical breathing and ICU admission in the event of pneumonia or acute respiratory distress syndrome (ARDS). The following factors were found to be substantially linked with death after ISN in pediatric COVID-19 patients: female gender, Asian ethnicity, admission to the ICU, mechanical ventilation, failure to get ISN reduction treatment (pneumatic or surgical), and suffering from ARDS. These results could aid in the development of focused interventions that increase the knowledge of medical professionals about the possibility of intestinal blockage in infants who visit the emergency room [38].

Despite being a common abdominal emergency, only 50% of infant intussusception cases exhibit the traditional clinical triad. The primary imaging method is ultrasound scanning, offering a 100% negative predictive value, an 88% specificity, and a sensitivity ranging from 98% to 100%. In emergency situations, plain films are necessary to screen for other illnesses or potential intestinal perforations. In cases requiring a surgical approach due to perforation, shock, or peritonitis, nonsurgical reduction (NSR) is employed to identify intussusception. Various contrast media, such as saline, air with fluoroscopic guidance, and barium suspension, are utilized. NSR proves to be a robust method compatible with any contrast media, applied in over 90% of cases [39].

Five instances of reduction failure were successfully resolved by switching to surgical reduction; these included one case of jejunoileal, 10% of ileoileal, and 9% of colocolic types; ileocolic types account for most cases. During intraoperative probing, which measured 1.5 cm in length from the ileocecal to the proximal intestinal canal, no significant abnormalities were discovered. Of the 100 patients, two experienced recurrences of disease within a year; among the surgically repaired patients, two patients had wound infections, and one patient developed paralytic ileus, which was managed conservatively [19].

## Conclusions

In cases of intussusception involving hemodynamically stable children without serious illness, pre-reduction antibiotics are deemed unnecessary. The optimal approach is to prioritize nonoperative outpatient therapy, with the option of employing minimally invasive procedures to avoid the need for laparotomy. Colonic intussusception should be removed in one piece, while enteric intussusception can be effectively treated through reduction followed by resection. Given the existence of intermediate forms of intussusception between colonic and enteric variations, a targeted approach is recommended. This recognizes the nuanced nature of intussusception and tailors treatment accordingly. Importantly, the standard treatment for adult intussusception remains to be surgical intervention.
